# Microglia in Health and Disease: A Double-Edged Sword

**DOI:** 10.1155/2017/7034143

**Published:** 2017-09-10

**Authors:** Ana Raquel Santiago, Liliana Bernardino, Marta Agudo-Barriuso, Joana Gonçalves

**Affiliations:** ^1^Institute for Biomedical Imaging and Life Sciences (IBILI), Faculty of Medicine, University of Coimbra, Coimbra, Portugal; ^2^Center for Neuroscience and Cell Biology-Institute for Biomedical Imaging and Life Sciences (CNC.IBILI) Research Unit, University of Coimbra, Coimbra, Portugal; ^3^Association for Innovation and Biomedical Research on Light and Image (AIBILI), Coimbra, Portugal; ^4^Health Sciences Research Center, Faculty of Health Sciences, University of Beira Interior, Covilhã, Portugal; ^5^Departamento de Oftalmología, Facultad de Medicina, Universidad de Murcia and Instituto Murciano de Investigación Biosanitaria Virgen de la Arrixaca, Murcia, Spain; ^6^Coimbra Institute for Biomedical Imaging and Translational Research/Institute of Nuclear Sciences Applied to Heath (CIBIT/ICNAS), University of Coimbra, Coimbra, Portugal

Microgial cells are the resident immune cells of the central nervous system, comprising 5–10% of the glial cells in the brain [1]. These cells orchestrate fundamental processes for the development and function of the CNS. Microglia participate in neuronal development, in adult neurogenesis, and also in the modulation of synaptic transmission [2, 3]. Microglia are constantly surveying the parenchyma, and they detect changes in their microenvironment, contributing to the pathophysiology of several neurodegenerative diseases. This special issue aimed to give an overview of the current knowledge on the role of microglial cells and processes mediated by microglia during health and disease.

F. I. Baptista et al. investigated how elevated concentration of glucose and interleukin-1*β* (IL-1*β*) negatively affects the progression of diabetic retinopathy, the most common complication of diabetes. In this paper, the exposure to elevated glucose concentration, to mimic hyperglycemic conditions, upregulates IL-1*β* expression in retinal neural cell cultures, affecting microglial and macroglial cells in the retina. The authors also observed that IL-1*β* has an important role in retinal microglial activation and proliferation under diabetic-like conditions, and limiting IL-1*β*-triggered inflammatory processes may provide a new therapeutic strategy to prevent the progression of diabetic retinopathy.

The proinflammatory cytokines, such as IL-1*β* and tumor necrosis factor (TNF), are important inflammatory mediators in the CNS. To date, the role of microglial-derived TNF following spinal cord injury (SCI) is poorly understood, since the contribution of soluble TNF (solTNF) versus membrane-anchored TNF (mTNF) to tissue damage and functional recovery remains to be elucidated. D. G. Ellman et al. investigated the effect of solTNF and mTNF on SCI using genetically modified mice that express only mTNF. They showed that the absence of solTNF in mice does not affect lesion size and functional outcome after SCI, but TNF levels are significantly decreased within the lesioned spinal cord. These findings suggested that genetic ablation of solTNF does not affect lesion size and functional outcome after SCI.

After spinal cord injury, inflammatory stimulation and/or modification greatly improve the regenerative outcome in rodents. I. Bollaerts et al. revised the current knowledge on how acute inflammation is intertwined with axonal regeneration, an important component of CNS repair.

Other severe motor neuron disease is amyotrophic lateral sclerosis (ALS), and C. Parisi et al. reviewed the M1/M2 functional imprinting of primary microglia as a paradigm of pro-/anti-inflammatory function and the role played by P2X7 and miR-125b in microglia activation in ALS. The authors concluded that a subtle equilibrium in the timing and power of proinflammatory versus anti-inflammatory agents can imprint microglia to tip the balance toward toxicity or protection, motor neuron survival, or cell death in ALS.

The balance between proinflammatory versus anti-inflammatory agents is crucial in several neurodegenerative disorders. Accordingly, D. Leonoudakis et al. explored the protective mechanisms of securinine, a major natural alkaloid product from the root of the plant *Securinega suffruticosa*, in glial cells. The authors demonstrated that this natural product inhibits glial activation and subsequent generation of proinflammatory factors.

Several agents have been reported to afford neuroprotection through the control of microglial reactivity. M. H. Madeira et al. revised the literature regarding the main effects of caffeine, the major component of coffee and the most consumed psychostimulant in the world, in the modulation of microglial reactivity and neuroinflammation in neurodegenerative diseases. Also, L. Carniglia et al. summarized the current literature on the way several neuropeptides modulate microglial activity and response to tissue damage and how this modulation may affect pain sensitivity.

It has been increasingly recognized that glial cells, such as microglia, and inflammatory signaling play a major role in the pathogenesis of chronic pain. T. Berta et al. revised the major signaling pathways involved in microglial cell activation and chronic pain with an emphasis on caspases. Overall, they suggested that caspase-6 released from axonal terminals regulates microglial TNF secretion, synaptic plasticity, and chronic pain. Because of this, they hypothesized that caspase-6 could be targeted by antibodies to treat chronic pain.

Together, the reviews and research articles that are included in this special issue help to understand the role of microglial cells in health and disease.

*Ana Raquel Santiago* *Liliana Bernardino* *Marta Agudo-Barriuso* *Joana Gonçalves*
